# Dysregulations of Expression of Genes of the Ubiquitin/SUMO Pathways in an In Vitro Model of Amyotrophic Lateral Sclerosis Combining Oxidative Stress and SOD1 Gene Mutation

**DOI:** 10.3390/ijms22041796

**Published:** 2021-02-11

**Authors:** Audrey Dangoumau, Sylviane Marouillat, Roxane Coelho, François Wurmser, Céline Brulard, Shanez Haouari, Frédéric Laumonnier, Philippe Corcia, Christian R. Andres, Hélène Blasco, Patrick Vourc’h

**Affiliations:** 1 UMR iBrain, Université de Tours, Inserm, 37000 Tours, France; audrey.dangoumau@gmail.com (A.D.); sylviane.marouillat@univ-tours.fr (S.M.); coelho.roxane@gmail.com (R.C.); francois.wurmser@gmail.com (F.W.); shanez.haouari@etu.univ-tours.fr (S.H.); frederic.laumonnier@univ-tours.fr (F.L.); philippe.corcia@univ-tours.fr (P.C.); andres@med.univ-tours.fr (C.R.A.); helene.blasco@univ-tours.fr (H.B.); 2 UTTIL, CHRU de Tours, 37000 Tours, France; C.BRULARD@chu-tours.fr; 3 Service de Neurologie, Centre de Référence sur la SLA, CHRU de Tours, 37000 Tours, France; 4 Service de Biochimie et de Biologie Moléculaire, CHRU de Tours, 37000 Tours, France

**Keywords:** ALS, ubiquitin, SUMO, SOD1, oxidative stress

## Abstract

Protein aggregates in affected motor neurons are a hallmark of amyotrophic lateral sclerosis (ALS), but the molecular pathways leading to their formation remain incompletely understood. Oxidative stress associated with age, the major risk factor in ALS, contributes to this neurodegeneration in ALS. We show that several genes coding for enzymes of the ubiquitin and small ubiquitin-related modifier (SUMO) pathways exhibit altered expression in motor neuronal cells exposed to oxidative stress, such as the CCNF gene mutated in ALS patients. Eleven of these genes were further studied in conditions combining oxidative stress and the expression of an ALS related mutant of the superoxide dismutase 1 (SOD1) gene. We observed a combined effect of these two environmental and genetic factors on the expression of genes, such as Uhrf2, Rbx1, Kdm2b, Ube2d2, Xaf1, and Senp1. Overall, we identified dysregulations in the expression of enzymes of the ubiquitin and SUMO pathways that may be of interest to better understand the pathophysiology of ALS and to protect motor neurons from oxidative stress and genetic alterations.

## 1. Introduction

Amyotrophic lateral sclerosis (ALS) is one of the most common adult-onset neurodegenerative diseases, caused by the selective death of motor neurons. A common feature in both sporadic ALS and familial ALS is the presence of protein aggregates rich in ubiquitin/ubiquitin-like proteins in motor neurons. These observations support a role for the ubiquitin proteasome system and ubiquitin-like small ubiquitin-related modifier (SUMO) system in ALS physiopathology [1,2,3,4].

Age is the major risk factor in the development of ALS [5]. Accumulative oxidative damages with age induce metabolic alterations, protein aggregation, and reduced mitochondrial function [6]. Oxidative damages are a consequence of an imbalance between a production of reactive oxygen species (ROS) and the ability of motor neurons/glial cells to reduce the level of ROS. Evidence of oxidative damages in ALS includes protein, lipid, and DNA oxidation observed in spinal cord and cerebrospinal fluid from ALS patients [7,8,9,10,11]. 

Current knowledge supports the involvement of interacting environmental and genetic factors in ALS. Superoxide dismutase 1 (SOD1) was the first gene identified in ALS [12]. More than 10% of the familial forms of ALS are linked to mutations in the SOD1 gene [13,14]. The mechanisms by which mutant SOD1 proteins cause cell death in patients are still unclear. Various mechanisms have been suggested, such as participation of oxidative stress, abnormal protein aggregation rich in ubiquitin/ubiquitin-like proteins, and disrupted axonal transport and mitochondrial dysfunction [15,16,17,18].

We present an analysis of gene expression in motor neuronal-like cell line NSC-34 exposed to oxidative stress. We next focus on genes of the ubiquitin (Ub)/Ub-like pathways and study their expression in cells exposed to both oxidative stress and an ALS-linked SOD1 mutant protein. 

## 2. Results

### 2.1. Changes in Gene Expression in NSC-34 Cells Exposed to Oxidative Stress

**Figure 1:**
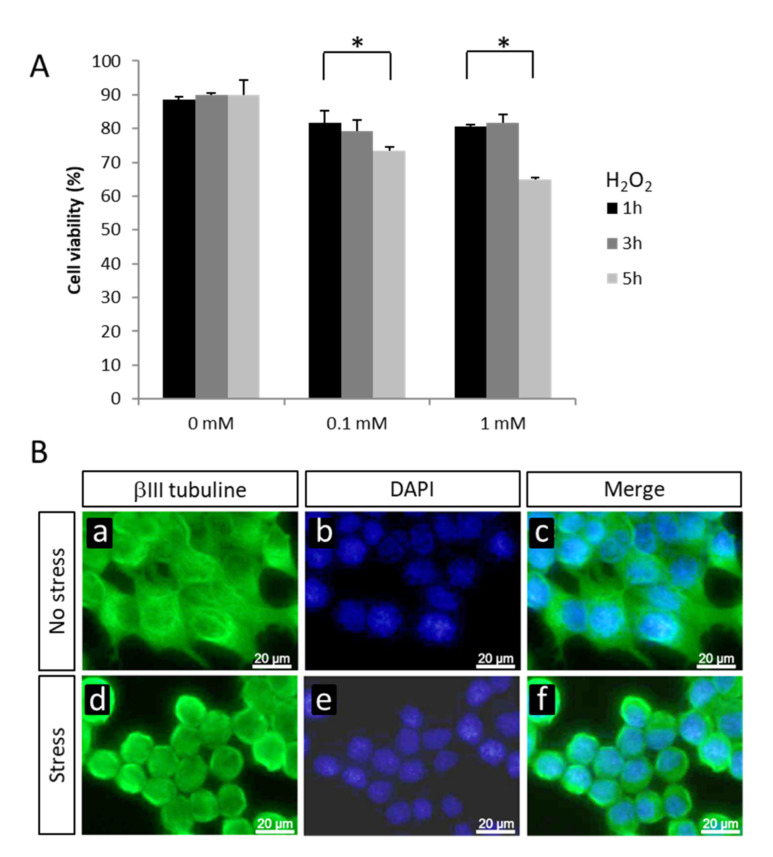
(**A**) Cell viability of non-transfected NSC-34 cells in absence or presence of 0.1 or 1 mM H_2_O_2_ for 1, 3, or 5 h. * *p* < 0.05 nonparametric Mann–Whitney test. (**B**) Immunocytochemical visualization of βIII-tubulin in NSC-34 cells in absence (**a**,**b**,**c**) or presence (**d**,**e**,**f**) of 1 mM H_2_O_2_ for 3 h. Scale bar: 20 µm.

Oxidative stress has been shown to contribute to motor neuron injuries in ALS. We developed an in vitro model using the mouse motor neuronal cell line NSC-34 exposed to the microenvironmental stress hydrogen peroxide (H_2_O_2_). NSC-34 cells exposed for 3 h to 0.1 or 1 mM H_2_O_2_ showed round-stressed shape without modification in cell viability (Figure 1A,B). Five hours after addition of 0.1 or 1 mM H_2_O_2_, a significant decrease in cell viability was observed (reduction by 18.3% and 28%, respectively; *p* < 0.05) (Figure 1A). We chose the 1 mM H_2_O_2_ condition for 3 h for the following gene expression study by microarray analyses.

Microarray analyses were performed with RNA samples from NSC-34 cell cultures expressing wild type human SOD1 in absence or presence of 1 mM H_2_O_2_ for 3 h. We used SurePrint G3 Mouse GE 60 K arrays (Agilent, Santa Clara, CA, USA), which contain 60,000 probes covering 39,430 Entrez gene RNAs and 16,251 long-noncoding RNAs (lcRNAs). Statistical analysis identified 360 probes associated with differentially expressed transcripts (*p* < 0.05) in cells exposed to oxidative stress, compared to cells cultured in normal condition. A total of 191 probes were linked to upregulated genes, and 169 probes to downregulated genes with *p*-value < 0.05 and fold change ≥ 1.3 (Table 1). Functional classification of the corresponding transcripts identified several biological pathways relevant in a context of neurodegenerative disease. These include cell response to DNA damage stimuli, apoptotic processes, antioxidant response, synaptic functions, mitochondrial function, and endosomal and Golgi functions. For example, we observed a decrease in expression of the cytotoxic granule associated RNA binding protein 1 (Tia1; fold change −2.83) whose implication in ALS pathology was recently supported by in vitro and in vivo studies [19]. We also described increase expression of methallothionein 1 (Mt1; fold change +7.51), previously studied in oxidative stress mechanisms and of interest in neurodegenerative diseases such as ALS [20,21].

### 2.2. Gene Expression Variations in the Ubiquitin/Ubiquitin-like Pathways

The analysis of microarray data pointed out an enrichment in genes of the ubiquitin (Ub)/Ub-like pathways (*p* < 0.05). To accurately identify all differentially expressed genes of these pathways in our data, we first created a list of all genes known to be implicated in these pathways by combining data from the databases Kyoto Encyclopedia of Genes and Genomes (KEGG), Ensembl, DUDEdb, and the literature [22]. We generated a list of 729 genes, with a majority of E2 conjugating enzymes, E3 ligases, and peptidases. Using this list, we reanalyzed the microarray data and identified 24 genes of the Ub/Ub-like pathways differentially expressed (fold change ≥ 1.3) in NSC-34 exposed to stress vs. cultured in normal condition. These genes are presented in Table 2 according to their enzymatic function. We found an increased expression of 10 genes, such as E2 Ube2r2, Ube2c, and E3 Fancl, Uhrf2, Rnf121. We observed a decreased expression for 14 genes, such as E2 Ube2d1, E3 CCNF, Trim9, and Rnf31, and SUMO1/sentrin-specific peptidase 1 (SENP1). Interestingly, mutations in the CCNF gene were recently observed in ALS patients [23].

### 2.3. Combined Effect of Oxidative Stress and Mutant SOD1^A4V^

To go a step forward to model the molecular mechanisms of motor neuron injury in ALS, we next investigated changes in gene expression in the Ub/Ub-like pathways induced by a combined action of oxidative stress and ALS-related SOD1 mutation. Based on knowledge in the literature, i.e., participation in stress responses and in other mechanisms implicated in neurodegenerative diseases, such as endoplasmic reticulum (ER) stress response, apoptosis, or mitophagy, we selected six out of the 24 genes of these pathways identified by our microarray analysis. These genes were Ube2e1, Ube2d2, Trim9, Uhrf2, Rbx1, and Senp1. We also reanalyzed our microarray data searching for genes of the Ub/Ub-like pathways close to significance in a context of oxidative stress (0.05 ≤ *p* < 0.06). We hypothesized that some of these genes may reach significance when combining oxidative stress and genetic mutations, such as SOD1 mutation. We selected five genes in this list, based on knowledge of functions in the literature, i.e., Ube3b, Birc3, Xaf1, Kdm2b, and Siah2.

**Figure 2:**
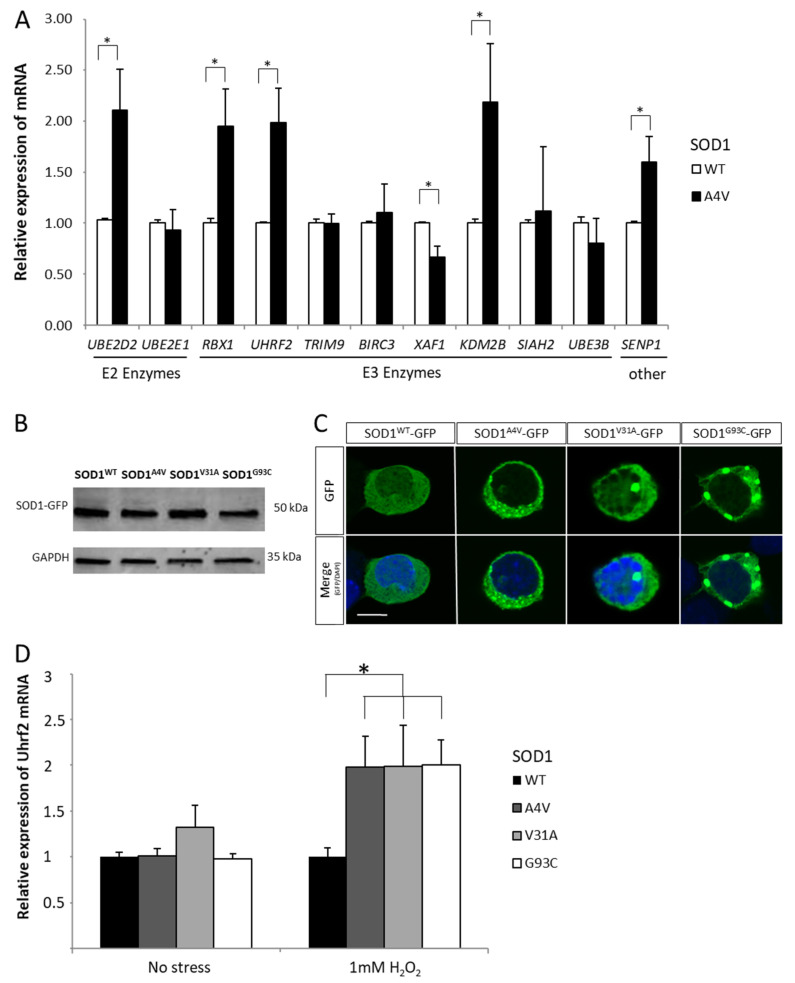
(**A**) Relative mRNA expression of Ube2d2, Ube2e1, Rbx1, Uhrf2, Trim9, Birc3, Xaf1, Kdm2b, Siah2, Ube3b, and Senp1 in NSC-34 cells expressing SOD1^WT^ or SOD1^A4V^ in presence of 1 mM of H_2_O_2_ for 3 h. (**B**) Western blotting of SOD1-GFP proteins in NSC-34 cells expressing SOD1^WT^-GFP, SOD1^A4V^-GFP, SOD1^V31A^-GFP, and SOD1^G93C^-GFP using antibodies against GFP or Gapdh. (**C**) Confocal microscopy analysis of SOD1^WT^–GFP or SOD1^mutant^-GFP in NSC-34 showing aggregates (48 h post-transfection). Scale bar: 30 µm. (**D**) Relative mRNA expression of Uhr2 in NSC-34 cells expressing SOD1^WT^, SOD1^A4V^, SOD1^V31A^, and SOD1^G93C^ in absence or presence of 1 mM of H_2_O_2_ for 3 h. * *p* < 0.05.

These 11 genes of the Ub/Ub-like pathways were analyzed by RT-qPCR in cultures of NSC-34 exposed to 1 mM H_2_0_2_ for 3 h and expressing the protein SOD1^WT^-GFP or the ALS-related mutant SOD1^A4V^-GFP. At time of exposure to oxidative stress, NSC-34 expressing SOD1^A4V^ for 48 h showed GFP positive aggregates in the cytoplasm (Figure 2). A combined effect of the ALS-related microenvironmental factor oxidative stress and an ALS-related genetic factor mutant SOD1^A4V^ affected significatively the expression of six out of the 11 genes selected in the Ub/Ub-like pathways (Figure 2). In a context of oxidative stress, NSC-34 expressing SOD1^A4V^ showed a significant increased expression of the E2 Ube2d2, the E3 Rbx1, Uhrf2, Kdm2b, and the peptidase Senp1 (*p* < 0.05). In contrast, the expression of Xaf1, an inhibitor of inhibitors of apoptosis proteins (IAPs), was significantly reduced (*p* < 0.05) [24].

### 2.4. Variation of Expression of the E3 Ligase Uhrf2 in Presence of SOD1 Mutants

To examine a possible participation of these differentially-expressed genes of the Ub/Ub-like pathway in motor neuron degeneration, we analyzed the consequence of the expression of various SOD1 mutants on one of these genes, the E3 ligase ubiquitin-like with PHD and RING finger domains 2 (Uhrf2), also known as Np95/ICBP90-like RING finger (NIRF). We focused on Uhrf2, a nuclear protein, because it is expressed in many regions of the CNS, including cortex, and it has been implicated in other neurodegenerative diseases with aggregate formation [25,26,27].

**Figure 3:**
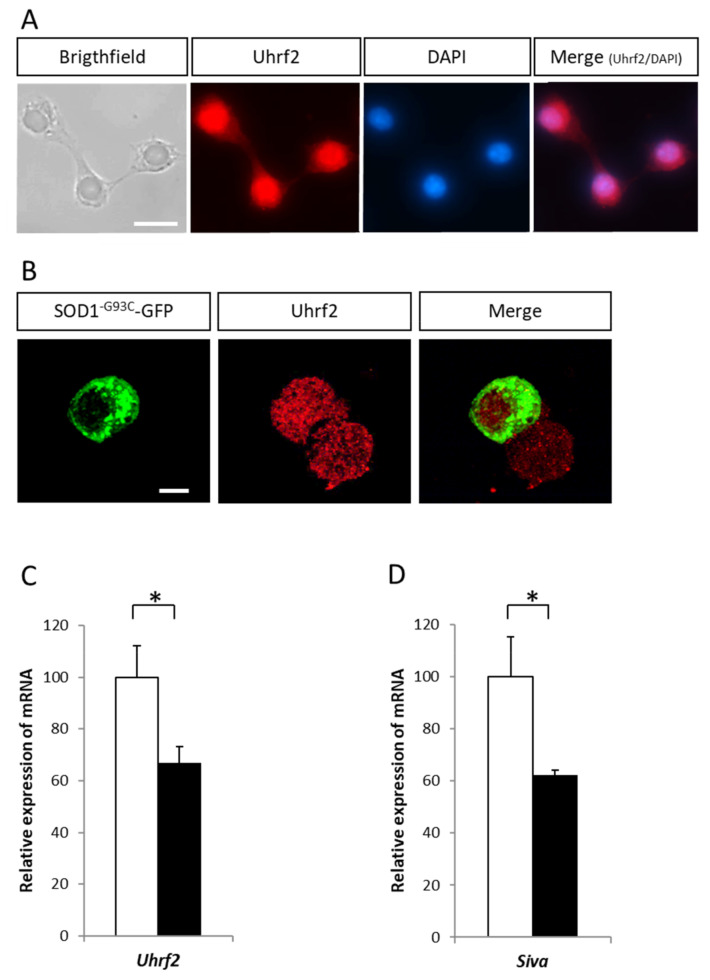
(**A**) Immunocytochemical visualization of Uhrf2 in NSC34 cells. (**B**) Absence of colocalization of SOD1^G93C^-GFP proteins with Uhrf2 in aggregates observed by confocal microscopy analysis. Scale bar: 10 µm. (**C**) Relative mRNA expression of Uhrf2 (RT-qPCR analysis) in NSC-34 cells coexpressing SOD1^G93C^-GFP and anti-Uhrf2 siRNA (or control siRNA) in presence of 1 mM of H_2_O_2_ for 3 h. (**D**) Relative mRNA expression of Siva in NSC-34 cells coexpressing SOD1^G93C^-GFP and siRNA anti-Uhrf2 (or siRNA control) in presence of 1 mM of H_2_O_2_ for 3 h (*n* = 3). * *p* < 0.05.

We first analyzed the effect of three ALS-related pathogenic SOD1 mutations (SOD1^A4V^, SOD1^V31A^, and SOD1^G93C^) on Uhrf2 gene expression. The three mutant SOD1s induced the formation of aggregates (Figure 2B,C) and an upregulation of Uhrf2 expression (Figure 2D). Uhrf2 protein was mainly nuclear (Figure 3A) and did not colocalize with SOD1^G93C^-GFP positive aggregates (Figure 3B, supplementary Figure S1A,B). We next examined the effect of siRNA-mediated reduction of Uhrf2 expression in NSC-34 cells expressing SOD1^G93C^-GFP for 24 h and exposed to oxidative stress for 3 h (1 mM H_2_O_2_) (Figure 3C). Uhrf2 binds to the promoter of the proapoptotic gene Siva in the U2OS osteosarcoma cell line [28]. In our model, siRNA-mediated reduction of Uhrf2 expression resulted in a significant decrease (37.6%) of Siva gene expression (Figure 3D). These data support a possible role of Uhrf2 in motor neuron degeneration observed in a context of ALS combining environmental and genetic factors.

## 3. Discussion

The present study was designed to analyze variations in gene expression that follow the exposure of NSC-34 cells, a motor neuronal cell line, to oxidative stress and to an ALS-related SOD1 mutant. A particular focus was given to members of the ubiquitin/ubiquitin-like systems [29,30].

We first investigated changes in global gene expression in NSC-34 cells exposed to oxidative stress. We chose to use NSC-34 cells because it is the most common cell line used in ALS research and it was described as having several morphological and physiological properties of motor neurons [31]. Moreover, NSC-34 cells allow high transfection rates, which is more difficult to obtain with other neuronal cell lines or primary cultures of neurons. However, these NSC-34 cultures are not primary cultures and are not in a glial environment, so the results will need to be confirmed in vivo. They represent a model of interest to investigate the mechanisms of motor neuron degeneration in ALS because they are not contaminated by astrocytes or other glial cells responsible for dilution effects in neuronal gene expression studies. Nevertheless, glial cells, and particularly astrocytes, are implicated in ALS pathogenesis, and thus their absence is a limit of our model. We obtained by microarray analysis a list of genes which, for some of them, have functions of interest in a context of neurodegenerative disease. A decrease in expression of T-cell intracellular antigen-1 (TIA-1) was observed. Tia1 is an mRNA binding protein that induces the formation of stress granules in the cytoplasm during cell stress [32]. It is a major component of stress granules, and similarly to other proteins located in stress granules, such as TDP-43, FUS, SMN1/2, and ATXN2, it could be of particular importance in ALS. Interestingly, immunoprecipitation experiments in NSC-34 cells showed that Tia-1 interacts directly with mutant SOD1 [19]. Increase in expression of methallothionein 1 (MT-1) was also observed. This is of particular interest because a recent study reported that overexpression of MT-1 extends lifespan in a mouse model of amyotrophic lateral sclerosis caused by mutant SOD1 [33]. MT-1 is one of the two major isoforms of MTs that exert neuroprotection against copper dyshomeostasis [34].

Interestingly we observed an enrichment of genes of the ubiquitin (Ub)/Ub-like pathways in our list of differentially expressed genes. Trim9 was one of the genes showing an important decrease in expression when motor neuronal cells were exposed to oxidative stress. The expression of this brain-specific E3 ubiquitin ligase Trim9 is decreased in aged mice, and is repressed in affected brain areas of Lewy body disease [35,36]. The expression of Ube2d2 (also known as UbcH5d), the E2 conjugating enzyme collaborating with the E3 Trim9, was also affected when NSC-34 were exposed to oxidative stress. The only function known for Trim9 is a ligase activity promoting SNARE-mediated vesicle fusion and axon branching [37]. SNARE-dependent exocytose participates in SOD1^G93A^ astrocytes-mediated toxicity in ALS [38]. On the contrary, Rbx1 gene was one of the genes showing an increase of expression during oxidative stress. It encodes the RING-box protein, a member of the SCF ubiquitin ligase complex controlling the degradation of the transcription factor nuclear factor-like 2 (Nrf2) in cells in basal conditions. In response to oxidative stress, Nrf2 translocates to the nucleus, controls the expression of a series of genes encoding detoxifying enzymes, anti-apoptotic proteins, and proteasomal proteins, and then is exported outside the nucleus for degradation [39]. Several studies support that NRF2 activation has a protective role against oxidative stress and cell death promoted by SOD1 mutations [40,41]. We also observed an increase of expression of Ube2r2 and Fancl, two genes of the Ub pathway implicated in the Wnt/β-catenin pathway. Accumulating evidence indicated that dysregulation of Wnt/β-catenin signaling is associated with neurodegenerative disorders [42]. A recent study showed that NSC-34 cells expressing mutated SOD1 show modifications of transcriptional activity associated with the Wnt/β-catenin pathway [43]. It is also very interesting to note that exposure of NSC-34 to oxidative stress modifies the expression of the CCNF gene encoding cyclin-F, which has recently been shown to be mutated in ALS patients [23]. CCNF could affect the activity of the VCP protein in the cytoplasm and participate in the formation of TDP-43 positive aggregates in patients when it is mutated [44].

Combined effects of microenvironmental factors such as oxidative stress and genetics factors such as mutation in SOD1 gene are highly suspected in the development of ALS. Based on our results, we decided to focus on genes of the Ub/Ub-like pathways, and particularly on 11 genes out of the 729 genes of the Ub/SUMO pathways. We selected three mutants, SOD1 A4V, G93C, and V31A, because they have all been reported in the literature (dominant mutations), are mutated at different positions in the protein SOD1, and are all associated with the formation of protein aggregates [45,46,47]. A4V is a mutant causing a rapidly progressive form of the disease, so we chose this mutant for RT-qPCR studies. We then selected G93C and V31A, two mutants associated with slower progressive forms of the disease. We first expressed the ALS-related mutant SOD1^A4V^ in NSC-34 cells and exposed these cells to 1 mM H_2_O_2_. We observed a combined effect on the expression of six genes encoding a E2 enzyme, four E3 enzymes, and one peptidase SENP. Some of them, the ubiquitin-E2, Ube2d2, and the SUMO-peptidase Senp1, can be directly linked to p53 activation, which plays a major role in the pathological process of motor neuron death in ALS [48,49,50,51]. In contrast to previous studies on spinal cord or primary cortical neurons from transgenic SOD1^G93A^ mice, we did not observe in our in vitro model an increase of expression of Ube2i, the unique E2 enzyme of the SUMO pathway [52,53]. This increase of expression of Ube2i could result from changes of expression in astrocytes present in these two models, and not in motor neurons. Glial cells were absent in our cultures [53].

The ubiquitin-like with PHD and RING finger domains 2 (Uhrf2) is another gene of interest. Also called Nirf (Np95/ICBP90-like RING finger), it encodes a ubiquitin-E3 ligase expressed in human brain [26]. UHRF2 is required for the proapoptotic activity of E2F1 transcription factor, as well as for the transcription of several genes encoding regulators of apoptosis [28]. Interestingly, a recent study showed that UHRF2 is also a SUMO-E3 ligase implicated in the SUMOylation of the transcription factor zinc finger protein 131 (ZNF131) highly expressed in the central nervous system and whose activation could have neuroprotective effect on motor neurons [54,55,56]. We showed that siRNA against Uhrf2 expression reduced the expression of Siva, an apoptosis-selective p53 target gene implicated in cell death [57]. Further studies will be necessary to show its direct involvement in ALS.

In summary, this gene expression study supports the idea that dysregulations in the Ub/SUMO pathways could affect protective mechanisms and trigger cell death mechanisms responsible for motor neuron degeneration in ALS. To our knowledge, this is the first global gene expression study that has attempted to analyze changes in these pathways in motor neuronal cells combining microenvironmental oxidative stress and ALS-related mutations in SOD1 gene. We showed additive effects of the oxidative stress and SOD1 mutations for particular genes of the Ub/SUMO pathways. We identified several genes of interest for further analysis through genetic studies in ALS populations or functional studies to identify new causative genes, or protective or risk factors for ALS.

## 4. Methods

### 4.1. Cell Cultures

NSC-34 cells were cultured at 37 °C in a 5% CO_2_ incubator in DMEM containing 25 mmol/L glucose, 0.58 g/L L-glutamine and 10% fetal bovine serum, 100 U/mL penicillin, and 100 µg/mL streptomycin (Sigma-Aldrich^®^, St. Louis, MO, USA). For oxidative stress conditions, cultures were treated with various concentrations of H_2_O_2_ for 1, 3, or 5 h.

### 4.2. Cell Viability Assay

For the Trypan blue assay, 48 h post-transfection the cells were trypsinized and centrifuged for 5 min at 2500× *g* and resuspended in DMEM, before addition of Trypan blue (0.4% *w*/*v*) (Molecular Probes-Invitrogen, Carlsbad, CA, USA). Living and dead cells were accurately counted by a Countess Automated Cell Counter (Invitrogen, Carlsbad, CA, USA).

### 4.3. Microarray Experiments

Total RNAs of NSC-34 cell line cultures were prepared using TRIzol^®^ extraction method (Invitrogen) and further purified using the RNeasy Micro Kit (Qiagen, Hilden, Germany). RNA quality was assessed by ND-1000 spectrophotometry (NanoDrop Technologies, Wilmington, DE, USA). Samples from four biological replicates of NSC-34 cell line transfected with human SOD1^WT^ plasmid and cultured in absence or presence of 1 mM H_2_O_2_ for 3 h were hybridized on SurePrint G3 Mouse GE 8 × 60 K Microarrays (Agilent).

The one-color microarray-based gene expression analysis protocol (version 5.7 of the One-Color Microarray-Based Gene Expression Analysis) was used to construct and to fluorescently label cRNA from total RNA extracts. Briefly, probes were prepared by converting an aliquot of 100 ng total RNA from each sample into labeled cRNA, using reagents from the one-color spike-in kit (Agilent Technologies) and one-color Quick amp labeling kit (catalogue No.5190-0442, Agilent Technologies). Total RNA were reverse transcribed into first and second-strand cDNA, after which first-strand cRNA were synthesized using the second-strand cDNA as a template in the presence of Cy3-CTP. Labeled cRNA were purified using the RNeasy mini spin column kit (Qiagen). Labeling efficiency was determined at 550 nm (Cy3) by ND-1000 spectrophotometry (NanoDrop Technologies).

Then, 800 ng Cy3-labelled cRNA were hybridized on individual 60 K arrays for 17 h at 65 °C. After hybridization, arrays were washed with Gene Expression Wash Buffers 1 and 2 (Agilent). Slides were scanned immediately following washing in the Agilent Scanner (SureScan, Plano, TX, USA) using Scan Control 9.1.7.1 software. The scan resolution was 3 µm. Scan data were extracted with Feature Extraction 10.7.3.1 software using the GE1_107 _Sep09 protocol. Extracted signal intensities were analyzed using GeneSpring 12.0 software (Agilent) and the datasets were normalized using standard Agilent FE Import 1-color settings. Genes were tested for differential expression using student tests, and *p*-values were corrected for multiple testing Benjamini and Hochberg; FDR 0.05. Analysis of cellular pathways was done using The Database for Annotation, Visualization and Integrated Discovery (DAVID) v6.7 [58].

### 4.4. Expression Vectors and Transfections

Full-length human wild type SOD1 cDNA was obtained using reverse transcription polymerase chain reaction on total RNA from leukocytes of a normal human individual, before insertion into pcDNA6.2/C-EmGFP-Gw/TOPO (Invitrogen, Carlsbad, CA, USA). Plasmids were amplified in TOP10 *Escherichia coli* bacteria, purified, and sequenced. Three ALS-related SOD1 mutants were generated by site-directed mutagenesis; SOD1^A4V^, SOD1^V31A^, and SOD1^G93C^ [45,46,59]. A total of 24 h after plating NSC-34 cells on poly-D-lysine coated surface (25 µg/mL), transfection of cells by plasmids (1.6 μg) or siRNA (1.6 μg, Silencer^®^Select anti-Uhrf2, s99417; Silencer Select negative control, Ambion^®^) was conducted with Lipofectamine 2000 according to manufacturer’s instructions (Invitrogen).

### 4.5. Western Blot Analysis

NSC-34 cells were lysed in Radio Immunoprecipitation Assay (RIPA) (ThermoFisher) buffer with protease inhibitors (Halt Protease Inhibitor Cocktail, Thermo Scientific). Then, 30 µg of proteins were separated by SDS-PAGE in a 4–15% polyacrylamide gel and transferred to a polyvinylidene fluoride (PVDF) membrane. The membrane was treated with 5% of milk in Tris-buffer containing 0.1% Tween-20 before overnight incubation at 4 °C with a polyclonal goat anti-GFP antibody (sc-5385, 1/200 Santa Cruz Biotechnology^®^ Inc., Dallas, TX, USA). Horseradish peroxydase-conjugated rabbit anti-goat antibody (81-1620, 1/2500, Zymed, South San Francisco, CA, USA) was used as secondary antibody before chemiluminescence analysis using ECL (Pierce-Thermo Fischer Scientific Inc., Rockford, IL, USA) and quantification by QuantityOne software (BioRad, Hercules, CA, USA). GAPDH expression was used for normalization using polyclonal goat anti-GAPDH antibody (sc-48166, 1/250, Santa Cruz Biotechnology Inc.) and secondary donkey anti-goat antibody (V8051, 1/2500, Promega, Madison, WI, USA).

### 4.6. Immunocytochemical Analysis

Cells were fixed with 4% paraformaldehyde (Sigma-Aldrich^®^) in phosphate buffer saline (PBS) for 45 min at room temperature (RT). After 1 h at RT in 1% bovine serum albumin, 5% horse serum, and 0.2% triton X-100 in PBS, cells were incubated overnight at 4 °C with mouse polyclonal antibody against β-3-tubulin (1:200, PRB-435P, Covance, Princeton, NJ, USA), or a polyclonal goat anti-UHRF2 antibody (1:200, sc-54252, Santa Cruz Biotechnology^®^ Inc.). This was followed by an incubation of 1 h at RT with FITC labeled antibody against mouse IgG (Jackson Immuno Research, West Grove, PA, USA, 115-095-205) or Fluoprobes 594 Donkey antibody against goat IgG (Interchim FP-SD2110). Preparations were mounted with Prolong Gold Antifade (Invitrogen^TM^) and observed under AMG Evos F1 microscope or Olympus Fluoview 500 confocal laser scanning microscope.

### 4.7. RT-qPCR Analysis

Total RNA were extracted from NSC-34 cells 48 h after transfection by plasmid expressing SOD1^WT^ or SOD1^mutant^, using Trizol (Invitrogen). Then, 1 μg RNA were treated with 1 μL RNase-free DNase I (Invitrogen). cDNA were synthesized using the SuperScript™II RT kit (Invitrogen), and qPCR was performed using 50 ng cDNA as template with SsoAdvanced™ SYBR^®^ Green Supermix (BioRad) reagent. Samples from four biological replicates were analyzed in duplicate in a LightCycler480 (Roche, Basel, Switzerland). The 2^−∆∆Cp^ method was used for quantifications. Normalized ratios were obtained for each target gene using the LC480 software (qPCR efficiencies for each gene were used), and *beta-actin* and *Gapdh* as normalization genes.

### 4.8. Statistical Analysis

Genespring (Agilent) and Excel software were used for statistical analysis. Data are presented as mean + − standard errors of the mean (SEM) of four independent experiments. Statistical analysis was performed using Student tests, and the non-parametric Mann–Whitney or Kruskall–Wallis tests.

## Figures and Tables

**Table 1:** Pathways of interest with genes differentially expressed in NSC-34 expressing superoxide dismutase 1 wild type (SOD1)^WT^ in presence of 1 mM H_2_O_2_ for 3 h (*p* < 0.05, fold change ≥ 1.3).

Gene Symbol			Gene Name	Fold Change
**Cellular response to DNA damage stimulus**				
*Cep164*	centrosomal protein 164			−1.80
*E2f7*	E2F transcription factor 7			−1.35
*Ube2b*	Ubiquitin-conjugating enzyme E2B			1.31
*Rbx1*	ring-box 1			1.35
*Brip1*	BRCA1 interacting protein C-terminal helicase 1			1.36
*Pcna*	proliferating cell nuclear antigen			1.37
*Fancl*	Fanconi anemia, complementation group L			1.47
*Gtf2h1*	general transcription factor II H, polypeptide 1			1.56
**Apoptotic process**				
Myc	myelocytomatosis oncogene			−2.83
*Tia1*	cytotoxic granule-associated RNA binding protein 1			−2.53
*Pde5a*	phosphodiesterase 5A, cGMP-specific			−2.21
*Mkl1*	MKL (megakaryoblastic leukemia)/myocardin-like 1			−1.82
*Bik*	BCL2-interacting killer			−1.71
*Dusp1*	dual specificity phosphatase 1			−1.65
*Pim1*	proviral integration site 1			−1.46
*Senp1*	SUMO1/sentrin specific peptidase 1			−1.34
*Ube2b*	Ubiquitin-conjugating enzyme E2B			1.31
*Tm2d1*	TM2 domain containing 1			1.38
*Birc5*	baculoviral IAP repeat-containing 5			1.41
*Mad2l1*	MAD2 mitotic arrest deficient-like 1			1.43
*Cfdp1*	craniofacial development protein 1			1.56
*Cycs*	cytochrome c, somatic			1.58
*Mt1*	metallothionein 1			7.51
**Antioxidant response**				
*Pdia2*	protein disulfide isomerase associated 2			−2.71
*Mtf1*	metal response element binding transcription factor 1			−1.63
**Synaptic functions**				
*Sipa1l1*	signal-induced proliferation-associated 1 like 1			−1.81
*Btbd9*	BTB (POZ) domain containing 9			−1.79
*Apba1*	amyloid beta (A4) precursor protein binding. family A, member 1			−1.79
*Nat8l*	N-acetyltransferase 8-like			−1.65
*Pacsin1*	protein kinase C and casein kinase substrate in neurons 1			−1.34
*Paip2*	polyadenylate-binding protein-interacting protein 2			1.55
*Cnn3*	calponin 3, acidic			2.25
**Mitochondrial function**				
*Bik*	BCL2-interacting killer			−1.71
*Alas1*	aminolevulinic acid synthase 1			−1.43
*Slmo2*	slowmo homolog 2 (Drosophila)			1.32
*Trit1*	tRNA isopentenyltransferase 1			1.35
*Metap1d*	methionyl aminopeptidase type 1D (mitochondrial)			1.36
*Lypla1*	lysophospholipase 1			1.38
*Hibadh*	3-hydroxyisobutyrate dehydrogenase			1.38
*Mrpl9*	mitochondrial ribosomal protein L9			1.41
*Pgam5*	phosphoglycerate mutase family member 5			1.42
*Timm8a1*	translocase of inner mitochondrial membrane 8A1			1.49
*Ndufab1*	NADH dehydrogenase (ubiquinone) 1, alpha/beta subcomplex, 1			1.54
*Lyrm7*	LYR motif containing 7			2.03
**Endosome and golgi functions**				
*Adcy6*	adenylate cyclase 6			−1.53
*Rab21*	RAB21, member RAS oncogene family			−1.35
*Rab9*	RAB9, member RAS oncogene family			1.33
*Pmel*	premelanosome protein			1.50
*Arl1*	ADP-ribosylation factor-like 1			1.52

**Table 2:** Gene of the ubiquitin (Ub)/Ub-like family differentially expressed in NSC-34 expressing SOD1^WT^ in presence of 1 mM H_2_O_2_ for 3 h (*p* < 0.05, fold change ≥ 1.3).

Gene Symbol	Gene Name	Fold Change
**E2 conjugating enzymes**		
*Ube2d1*	Ubiquitin-conjugating enzyme E2D1	−1.87
*Ube2d2*	Ubiquitin-conjugating enzyme E2D2	1.30
*Ube2b*	Ubiquitin-conjugating enzyme E2B	1.31
*Ube2e1*	Ubiquitin-conjugating enzyme E2E1	1.37
*Ube2c*	Ubiquitin-conjugating enzyme E2C	1.39
**E3 ligases**		
**E3 ligases with multipled subunits with RING domain**		
Subunits with RING domain		
*Rbx1*	Ring-box 1	1.35
Subunits (adaptators)		
*CCNF*	Cyclin F baisse	−2.44
*Spsb4*	SpIA/ryanodine receptor domain and SOCS box containing 4	−1.92
*Btbd9*	BTB (POZ) domain containing 9	−1.78
*Klhl29*	Kelch-like 29 (Drosophila)	−1.61
*Fbxo46*	F-box protein 46	−1.54
*Rhobtb2*	Rho-related BTB domain containing 2	−1.43
*Klhl21*	Kelch-like 21 (Drosophila)	−1.33
*Fbxo42*	F-box protein 42	−1.26
*Cdc16*	CDC16 cell division cycle 16 homolog (S.cerevisiae)	1.30
*Zbtb32*	Zinc finger and BTB domain containing 32	1.41
**other E3 ligases**		
*Trim9*	Tripartite motif-containing 9, transcript variant 3	−2.27
*Sh3rf1*	SH3 domain containing RING finger 1	−1.95
*Rnf121*	RING finger protein 121	1.35
*Fancl*	Fanconi anemia, complementation group L	1.46
*Uhrf2*	Ubiquitin-like, containing PHD and RING finger domains 2	1.49
**Others**		
*Usp44*	Ubiquitin specific peptidase 44	−2.25
*Usp36*	Ubiquitin specific peptidase 36	−1.70
*Senp1*	SUMO1/sentrin specific peptidase 1	−1.34

## Supplementary Materials

The following are available online at https://www.mdpi.com/1422-0067/22/4/1796/s1, Figure S1: (A) Localization of Uhrf2 protein in NSC-34 cells expressing SOD1^WT^-GFP (a, b) or SOD1^G93C^-GFP (c, d), in absence (a–c) or presence (b–d) of 1mM of H_2_O_2_ during 3 hours. Scale bar: 50 µm (B) Control goat IgG antibody. Scale bar: 50 µm
